# Immune Response and Transplacental Antibody Transfer in Pregnant Women after COVID-19 Vaccination

**DOI:** 10.3390/jpm13040689

**Published:** 2023-04-20

**Authors:** Chiara Lubrano, Alessandro Mancon, Gaia Maria Anelli, Gloria Gagliardi, Roberta Corneo, Micol Bianchi, Chiara Coco, Giulia Dal Molin, Michele Vignali, Irene Schirripa, Nicoletta Di Simone, Giulia Pavone, Antonio Pellegrino, Maria Rita Gismondo, Valeria Maria Savasi, Irene Cetin

**Affiliations:** 1 Department of Biomedical and Clinical Sciences, Università degli Studi di Milano, 20157 Milan, Italy; 2 Laboratory of Clinical Microbiology, Virology and Bioemergencies, ASST Fatebenefratelli Sacco, Luigi Sacco Hospital, 20157 Milan, Italy; 3 Department of Biomedical Science for Health, Macedonio Melloni Hospital-ASST Fatebenefratelli Sacco, University of Milan, 20133 Milan, Italy; 4 Department of Biomedical Sciences, Humanitas University, Pieve Emanuele, 20072 Milan, Italy; 5 IRCCS Humanitas Research Hospital, Rozzano, 20089 Milan, Italy; 6 Department of Woman, Child and Neonate, Luigi Sacco Hospital, ASST Fatebenefratelli Sacco, 20157 Milan, Italy; 7 Unit of Obstetrics and Gynecology, Alessandro Manzoni Hospital, ASST Lecco, 23900 Lecco, Italy; 8 Department of Woman, Mother and Neonate, Buzzi Children’s Hospital, ASST Fatebenefratelli Sacco, 20154 Milan, Italy

**Keywords:** COVID-19, SARS-CoV-2, immunization in pregnancy, humoral response, T-cell response, transplacental antibody transfer

## Abstract

COVID-19 infection is associated with increased risk of pregnancy complications, making vaccination during pregnancy critical for mother-neonate dyads. Few data, often with an unrepresentative sample size, are available on SARS-CoV-2 vaccine-induced humoral and cell-mediated response. Here, we evaluated anti-S antibody and interferon-gamma (IFN-γ) production elicited by SARS-CoV-2 immunization in maternal and neonatal plasma. Pregnant women (*n* = 230) were prospectively enrolled and classified as unvaccinated (*n* = 103) and vaccinated (*n* = 127); after serological screening for previous infections, assays were performed on 126 dyads, 15 mothers and 17 newborns. Positive anti-S antibodies were found in most of the vaccinated subjects, regardless of timespan between immunization and delivery (range: 7–391 days). A total of 89 of 92 vaccinated women showed a broad response to COVID-19 immunization and highly effective placental transfer, as attested by anti-S positive rates (maternal = 96.7%, cord = 96.6%). Most of our subjects had indeterminate results in an IGRA assay, preventing a conclusive evaluation of IFN-γ production. Indeed, pregnancy-related hormonal changes may influence T-cell response with an impact on IFN-γ production. Positive pregnancy and perinatal outcomes reinforce the evidence that the anti-SARS-CoV-2 immunization is effective and well-tolerated in pregnant women and also protective for the fetus/neonate, even though it was not possible to define the related IFN-γ production and role.

## 1. Introduction

Since the beginning of the COVID-19 pandemic, pregnant women have been considered a high-risk group. Several studies have showed an increase in severe maternal and obstetric complications after symptomatic COVID-19 infection, including admission to an intensive care unit, invasive ventilation, preeclampsia, preterm birth, and low birth weight [1,2,3,4]. Furthermore, infants are at significantly higher risk for serious COVID-19 compared with children [5].

It is known that vaccination during pregnancy, besides providing protection to the mothers, plays a major role in the prevention of neonatal infectious diseases, thanks to the transplacental transfer of maternal antibodies to the fetus [6,7]. A growing body of evidence underlines the efficacy of anti-SARS-CoV-2 immunization in eliciting an effective humoral response during pregnancy. Trostle et al. observed the presence of specific anti-spike protein (anti-S) antibodies in 100% of cord blood samples from pregnant women vaccinated with at least one dose of a vaccine, supporting neonatal seroprotection from the early stages of intrauterine life, when vaccination is currently not allowed [8]. Moreover, a review by Prasad et al. underlined that immunization did not negatively influence pregnancy outcomes by comparing vaccinated and unvaccinated cohorts. Indeed, vaccination protects against severe forms of SARS-CoV-2 infection, which in pregnancy are associated with intensive care unit admission, preterm delivery, preeclampsia and cesarean section [9,10]. Given this, and considering the widely demonstrated safety and effectiveness of the vaccine for both mother and fetus, national and international societies have strongly recommended SARS-CoV-2 vaccine administration to pregnant women [11,12,13,14,15]. Consequently, in July 2021 the vaccine recommendation was extended in Lombardy (Italy) to all pregnant women [16]. Nevertheless, hesitancy in undergoing immunization remains a significant issue in the pregnant population, typically ascribable to social and educational factors (i.e., source of information, graduation degree, family influence) [17,18,19].

Few data are currently available on the persistence and dynamics of antibodies and their transfer through the placenta after COVID-19 vaccination. Moreover, while immunoglobulin production reflects humoral immune response, interferon-gamma (IFN-γ) is a measure of antiviral proinflammatory T helper 1 (Th1) lymphocytes activity [20]. Indeed, cytokine release has also been demonstrated due to stimulation by SARS-CoV-2 antigens in both natural infections and passive immunization [21,22], and is suggested to play a crucial role in prolonged immune response, reinfection prevention and viral clearance [23,24,25]. Since immunomodulation at different gestational ages induces alteration in IFN-γ production, data on its kinetics are required to clarify its importance in fetal and maternal protection. In the context of COVID-19 diagnosis, ELISpot and QUANTIFeron are reliable and specific methods for assessing IFN-γ production upon stimulation by pathogen-specific antigens [26,27,28].

This study aimed to evaluate the seroconversion rate and effectiveness of transplacental transfer of antibodies in pregnant women after SARS-CoV-2 vaccination, through quantification of immunoglobulins in maternal and neonatal systemic circulation in relation to vaccine schedule. In addition, T-cell response was analyzed by measuring specific viral-antigen-stimulated IFN-γ release in maternal and cord plasma. Lastly, neonatal outcomes were compared between vaccinated and unvaccinated newborns.

## 2. Materials and Methods

### 2.1. Study Population

This is a multicenter prospective study conducted at the Obstetrics and Gynecology Departments of Luigi Sacco, Macedonio Melloni and Vittore Buzzi Children’s Hospitals (ASST Fatebenefratelli-Sacco, Milan), Pio X Humanitas Hospital (Milan), and Manzoni Hospital (Lecco), Italy. Pregnant women who underwent immunization against COVID-19 during or less than three months before pregnancy were enrolled, and unvaccinated women were selected as a control group. The enrolled pregnant women (April–November 2021) were also a subset of a larger Lombardy cohort that underwent a cross-sectional survey of sociodemographic/clinical characteristics and attitudes about COVID-19 vaccination [19].

The following exclusion criteria were used: no provision of informed consent; polyallergy or hypersensitivity to any vaccine component; previous SARS-CoV-2 infection; administration of any other immunization in the previous 14 days; progressive or uncontrolled neurologic syndromes; ongoing acute febrile syndrome; immunodeficiency or ongoing immunosuppressive regimen; HIV patients.

Previous SARS-CoV-2 infection was defined as a documented positive molecular or serological result before immunization; in addition, when no test was available, screening for anti-SARS-CoV-2 antibodies was performed on blood samples collected at enrollment. Unvaccinated subjects were tested for both anti-nucleocapsid (anti-N) and anti-S antibodies to overcome the limited persistence of former vaccines; in vaccinated individuals, only the anti-N test was performed, since anti-S antibodies are produced after immunization. All positive individuals were excluded.

Immunization response was evaluated based on the presence of anti-S SARS-CoV-2 antibodies in both maternal and cord serum samples.

In a subset of patients, cellular response was assessed using a specific Interferon Gamma Release Assay (IGRA). A sample size of at least 50 vaccinated and 100 unvaccinated women was estimated.

**Figure 1:**
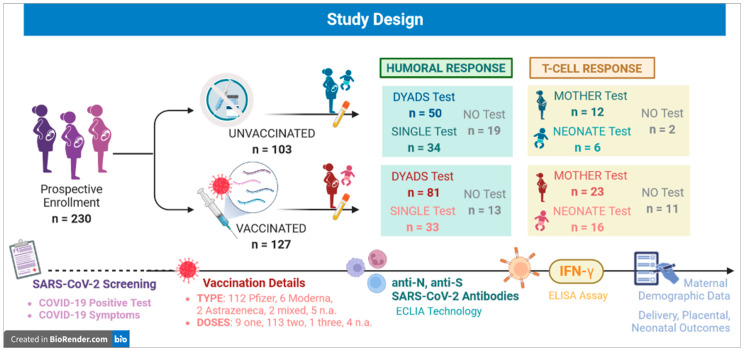
Study design.

The study design is presented in Figure 1.

Both humoral and t-cell response were tested on maternal and cord blood. No Test: samples were too low in volume, hemolyzed or coagulated to perform any test. Dyads: mother-neonate couples.

Demographic, pregnancy, clinically relevant and vaccine-related data were recorded.

All women filled out an anonymous questionnaire regarding social demographic characteristics and COVID-19 vaccine uptake [19].

The study was conducted in accordance with the Declaration of Helsinki and in compliance with all current Good Clinical Practice guidelines, local laws, regulations, and organizations. The protocol was approved by the Medical Ethics and Institutional Review Board (Comitato Etico Milano Area 1, No. 0032542); all participants gave their informed consent to collect personal data and biological samples.

### 2.2. SARS-CoV-2 Anti-N and Anti-S Antibody Measurement

Maternal and cord blood were collected and centrifuged for serological analysis.

The production of anti-N SARS-CoV-2 antibodies was assessed using the Elecsys Anti-SARS-CoV-2 kit (Roche Diagnostics, Rotkreuz, Switzerland), which targets all immunoglobulin classes with a positive cut-off of ≥1.0 COI (cut-off index).

Anti-S was measured with the Elecsys Anti-SARS-CoV-2 S kit (Roche Diagnostics, Rotkreuz, Switzerland), which targets RBD with a positive linear range of 0.8–250 unit/mL (U/mL).

Both tests were based on Electrochemiluminescent Immunoassay (ECLIA) technology and were run on the automated Cobas e 411 analyzer (Roche Diagnostics, Rotkreuz, Switzerland).

### 2.3. Anti-SARS-CoV-2 Evaluation of T-Cell Response

T-cell response was measured using the IGRA QuantiFERON (QNF) ELISA SARS-CoV-2 assay (QIAGEN, Hilden, Germany).

In brief, venous blood was collected in four test tubes: Ag1 (CD4+ stimulation), Ag2 (CD4+ and CD8+ stimulation), Mitogen (IFN-γ production control), and Nil (background IFN-γ control). Both Ag1 and Ag2 were coated with a mixture of viral S protein peptides to induce specific IFN-γ production. After a 16–24 h incubation at 37 °C, the tubes were centrifuged and the plasma was analyzed using a microplate of Enzyme-Linked Immunosorbent Assay (ELISA) for IFN-γ dosage according to the manufacturer’s instructions.

The data were interpreted with QuantiFERON SARS-CoV-2 software (v1.0.0, QIAGEN, Hilden, Germany), which classifies samples as reactive (presence of S-peptide-induced IFN-γ), non-reactive (absence of S-peptide-induced IFN-γ) or undetermined (inconclusive result).

### 2.4. Statistical Analysis

Maternal demographic, clinical, obstetrical, and immunological characteristics and delivery outcomes were analyzed in the full study population and then compared between immunization subgroups (vaccinated vs. unvaccinated pregnant women) using a Chi-square for independence (with Yates continuity correction) or exact (Fisher’s) tests for ordinal variables, and a Mann–Whitney U test or Student’s *t*-test for continuous variables.

Participants’ baseline characteristics were presented as frequencies for categorical variables or mean ± standard deviation for quantitative continuous variables.

Comparisons between groups and correlations were considered statistically significant when *p*-value < 0.05.

Statistical analyses were performed using the software SPSS, v.27 (IBM; Armonk, NY, USA).

## 3. Results

### 3.1. Study Population

A total of 230 pregnant women were enrolled and screened for anti-SARS-CoV-2 specific antibodies: none of them reported previous molecular or serological positive results or potential symptomatic infections.

Figure 1 shows the number of enrolled patients and of maternal and cord blood samples tested.

Table 1 summarizes patients’ demographic, anamnestic and clinical information.

Most subjects were Caucasian (191/230, 83.0%) and vaccinated (127/230, 55.2%).

Regarding vaccine administration, a total of 113 subjects completed a two-dose schedule, only 1 also had the booster dose, and 9 received the first vaccination at the time of enrollment. In addition, at least 4 weeks elapsed between immunization and sample collection in 74.4% of patients, a time span sufficient to detect immune response. In 23 of the 46 participants for whom data were available, adverse events occurred, namely, pain at the site of injection (18/46, 39.1%), fever (3/46, 6.5%), and headache (2/46, 4.3%).

Table 2 shows the comparison between vaccinated and unvaccinated women in terms of maternal characteristics and pregnancy outcomes.

Unvaccinated and vaccinated pregnant women had the same mean age despite the quite large range reported in Table 1. More than half (*n* = 134, ~59%) of our mothers were between 31–38 years old, representing the first and third quartile values, respectively.

Concerning ethnicity, only 6.3% of vaccinated subjects were non-Caucasian, while 93.7% were Caucasians. The same inequality is evident in the unvaccinated group, with 30.1% non-Caucasian vs. 69.9% Caucasian subjects (*p*-value: *p* < 0.001). In regard to maternal biometrics, pregestational Body Mass Index (BMI) was slightly higher in the unvaccinated women (*p* = 0.05).

Maternal parity did not differ between groups; the most common pregnancy-associated disease was gestational diabetes (*n* = 39, 17% of the study population), with a limited proportion of subjects affected by other comorbidities with comparable frequencies in vaccinated and unvaccinated subjects.

Only five preterm deliveries (<37 gestational weeks) were recorded in the study population, regardless of vaccination status.

Concerning placental features, unvaccinated mothers delivered heavier placentas (*p* = 0.02) with a lower neonatal/placental weight ratio (*p* = 0.001), which is usually indicative of lower placental efficiency. Neonatal weight and pH were similar between the two analyzed groups.

### 3.2. Serological Screening for Pre-Study Undiagnosed SARS-CoV-2 Infections

To exclude the influence of immune response elicited by past undiagnosed infections, the population was screened for anti-N and anti-S positive results as proxies for previous exposure to SARS-CoV-2.

Specimens from mother-neonate dyads were available for 50 unvaccinated and 81 vaccinated subjects, while single sera were available for 67 subjects, for a total of 198. For the remaining patients (32/230), specimens were too low in volume or coagulated.

The anti-N assay was positive in 16/84 (19.05%) unvaccinated and 16/114 (13.91%) vaccinated mothers and neonates. The anti-S assay performed in the unvaccinated population revealed 8/84 (9.52%) further pre-study infections, for an overall positive rate of 20.10% (Table 3).

The serological screening showed that approximately 20% of the analyzed population had a SARS-CoV-2 infection before study enrollment. Unvaccinated individuals were excluded when anti-N and/or anti-S antibodies were detected in maternal plasma, cord plasma or both; in the vaccinated group, only anti-N antibodies were considered, since anti-S antibodies are usually produced after passive immunization and may not be discernible from natural infections.

Ultimately, antibody production was evaluated in 284 samples: 126 dyads, 15 maternal-only and 17 cord-only blood samples, separated into vaccinated and unvaccinated pools. The former group included 81 couples, 11 maternal and 6 fetal samples; the latter one consisted of 45 dyads, 4 maternal and 11 fetal specimens (Table 4a). All samples of the unvaccinated population were negative (Table 4a), with complete concordance of paired mothers and cord blood when considering dyads (Table 4b).

Data on immunized subjects are discussed in the following section.

### 3.3. Humoral Response to Anti-SARS-CoV-2 Vaccination

Positive anti-S antibodies were found in 94/98 (95.9%) vaccinated subjects. Total Ig were detected in 78 out of 81 dyads, in all maternal-only (*n* = 11) and in 5/6 cord-only samples, with negative tests in the remaining 4 cases.

Results were then evaluated in relation to vaccination schedules to identify any possible influencing factor, especially in absence of Abs production. Most participants received the second dose (92/98, 93.9%), and only one received the third booster, while for two subjects no information was available. Almost all the mothers (82.8%) delivered at least in two weeks, and at most one month after the second dose (*n* = 68, 68.67%). No comparison was possible between different vaccines, since Cominarty (Pfizer) was administrated in 87 out of 98 women (88.8%) (Table 1).

In 60/78 positive dyads, a value of Abs > 250 U/mL was found in both maternal and cord samples; this was also true in five cases with an immunization–delivery interval < 14 days. Among the other 20 dyads, immunoglobulins titer was higher in 5/18 maternal and 13/18 cord specimens, respectively. Interestingly, maternal antibody concentration was higher within an after-dose temporal limit of 23 days in all but one case, while cord titer exceeded the maternal one at least 54 days after dose. Only one discordant occurrence was found, with a negative result for the mother sample and a value of >250 U/mL for the cord blood 21 days after the second dose.

In the maternal-only group, a value of 196.20 U/mL was the only <250 U/mL detected, still considered high. Regrettably, data about vaccination timing for this subject and two further cases are missing. For all the other subjects who received a second Pfizer dose, the range of the immunization–delivery interval was 38–322 days.

For the cord-only negative sample, no data about the vaccine schedule was available. Among positive samples, one had a 54.75 U/mL concentration of Abs 32 days after the first dose and all the others were >250 U/mL (time range 31–103 days).

Regarding negative findings, both maternal and cord specimens tested negative for the woman who had only one vaccine dose 14 days before blood collection. Another dyad had no evidence of seroconversion 6 days after the second dose, while for the third occurrence only cord blood was analyzed and no information on immunization was available.

### 3.4. Anti-SARS-CoV-2 T-Cell Response Evaluation

The IGRA test was performed on 35 maternal and 22 related cord plasma samples, 23 and 16 of which, respectively, belonged to the vaccination group. In addition, four subjects in each pool were identified as undiagnosed previous infections based on serological tests; data from these subjects were useful to search for possible differences in cellular response elicited by natural infection or immunization. The assessment of IFN-γ release was performed in a small subset of subjects who gave their consent to biological sample collection.

No induction of IFN-γ by viral antigens was found in 50.0% of the unvaccinated women, while the other 50.0% and all cord-blood samples were indeterminate (Table 5).

Most tests returned an indeterminate result, constraining the possibility of calculating statistically significant differences between the vaccinated and unvaccinated populations, or within the vaccinated group according to immunization schedule. Nevertheless, the only reactive result in the vaccinated group underlines the possible role of pregnancy condition in influencing T-cell mediated immune response (detailed results in Table S1).

## 4. Discussion

In this study we report a high seroconversion rate in pregnant women receiving SARS-CoV-2 vaccination: a total of 89/92 (96.7%) subjects demonstrated a response to COVID-19 immunization, based on the high titers of anti-S Abs detected.

Such evidence assumes great importance because it confirms the efficacy of vaccination in eliciting immune response during pregnancy, and paves the way for comparing the possible deleterious outcomes of complicated COVID-19. In 70 of 92 subjects, the Abs value was >250 IU/mL regardless of timespan between immunization and delivery (range: 7–391 days). In addition, the majority of the women (57.1%) received their last dose during the third trimester.

Previous studies have demonstrated that most uninfected individuals showed seroconversion at the time of second dose administration; the percentage of Abs-positive subjects increased rapidly at the next study follow-up [29,30,31].

The present work showed how this observation can also be extended to pregnant population, considering that 85.7% of samples were collected after the second dose. Indeed, only one subject who received a single dose had not shown seroconversion 14 days after administration. These data further support the effectiveness of immunization in the third trimester. A previous study showed increased immunogenicity in the third trimester, likely related to enhanced anti-S Abs functionality and ability to bind to the Fc Receptor (FcR) despite the lack of difference in titers [32]. The same observation was made by Prabhu et al., who found a comparable response to vaccination among trimesters, and even more interestingly, between pregnant and nonpregnant women [33].

From an epidemiological point of view, 20.10% of the study population experienced an undiagnosed SARS-CoV-2 infection; none of the participants declared a previous positive molecular, antigenic nor serological test, while anti-N and anti-S Abs presence was detected in sera collected at enrolment. This evidence could also be underestimated. It was shown that the anti-N Abs titer declines more rapidly over time compared to anti-S, especially in patients presenting asymptomatic or mild disease who also reported lower Ig concentrations [34,35,36]. In our cohort, anti-S antibodies were found in only eight sera from unvaccinated subjects; we were not able to evaluate the same data in vaccinated subjects due to the overlap with vaccine-induced response, which prevented a more precise estimation. In this respect, a recent (September 2021) meta-analysis by Bergeri et al. estimated global infection-attributable seroprevalence of 35.9%, exceeding the concomitant number of reported COVID-19 cases [37].

Concerning maternal clinical data, the two analyzed groups did not show any difference. Delivery outcomes appeared similar regardless of whether vaccination occurred, with no adverse events reported. In terms of placental features, unvaccinated women delivered heavier and less efficient placentas (i.e., decreased neonatal/placental-N/P ratio) than vaccinated mothers [38]. The N/P ratio is often used as a proxy for placental efficiency [39]. A decreased N/P ratio is commonly associated with maternal obesity, diabetes, and preeclampsia [40,41,42]. However, this study population showed further differences in terms of vaccination in maternal clinical features (e.g., maternal ethnicity and BMI), although the statistical significance was low. Thus, there is likely to be a multifactorial influence on placental features such as weight and efficiency, possibly unrelated to COVID-19 vaccination.

No difference concerning neonatal outcomes was recorded; newborns from unvaccinated and vaccinated women showed similar neonatal weight, centiles, and umbilical artery pH. This reinforces the evidence that vaccination does not lead to increased inflammation or related pregnancy complications, confirming its safety in pregnancy.

Apart from the optimal maternal response, a main issue of interest is placental antibody transfer to ensure fetal and newborn protection against SARS-CoV-2 infection [43]. IgG are the main actors, since they usually cross the placental barriers using the FcRs on the syncytiotrophoblast as transporters. Antibody transfer seems to be active starting in the first trimester with progressive increase over time, reaching a peak in the period from the 28th gestational week until the end of pregnancy, probably due to the increased FcR expression [44]. In our population, all but one of the cord blood samples were anti-S positive, usually with a concentration > 250 U/mL, suggesting highly effective placental transfer. This was also true in the few cases with administration of a vaccine during the first trimester or even before pregnancy.

However, in a limited number of dyads, a difference in the last dose–delivery interval was observed when comparing maternal and neonate dyads’ Abs titers (<250 U/mL). Interestingly, maternal IgG values were higher up to a 23-day interval, while the opposite scenario was present after 54 days. These data suggest an inverse Abs dynamic overtime, with parallel decay opposite to an increase in maternal and fetal blood, respectively. In the abovementioned study by Prabhu et al., the anti-S IgG reduction was shown for both pregnant and nonpregnant females with no significant difference detected during the follow-up, even 6–8 months after immunization. In addition, maternal and cord Abs levels appeared to be strictly correlated [33,45]. High rates of successful seroconversion and placental transfer of immunoglobulins were reported in pregnant women on different vaccine platforms, underlying a higher cord blood titer when vaccination was performed in the first or second trimester [46]. The time-related waning of immune response represents a significant concern for pregnancy, as the loss of maternal protection could affect the fetus. This observation raises the question of administering booster doses in this group to minimize adverse outcome in the perinatal period, as suggested by Faust et al. [47]. Indeed, additional doses would provide a response against new emerging variants [48,49].

Regarding the evaluation of specific anti-SARS-CoV-2 IFN-γ production, most tests had indeterminate or negative results in both mothers and fetuses. The IGRA assay was performed in a small proportion of subjects, making it difficult to obtain clear indications on cell-mediated response. However, our data could be explained by the changes characterizing the immune system during pregnancy. Hormones, cell populations and cytokines expression undergo extensive modulation to allow fetal tolerance [50]; in particular, IFN-γ seems to be actively released in the first trimester to help with uterus remodeling and implantation, while its expression is downregulated in the second and third trimesters [51]. In the context of the IGRA assay, the IFN-γ downregulation is detected as an indeterminate result, meaning that the subject had impaired cytokine production per a negative mitogen test. Specific anti-SARS-CoV-2 IFN-γ release was reported in the general population after vaccination, as well as in pregnant women experiencing COVID-19, especially when experiencing severe disease [52,53,54]. In addition, Collier et al. found comparable cytokine production in nonpregnant, pregnant, and lactating women, although this did not correlate with vaccination timing [55].

Although it was not the aim of the present work, immunization in our population appeared safe and well tolerated, with only mild adverse effects registered, even when a previously undiagnosed SARS-CoV-2 infection was detected by serological tests. Furthermore, no increment was detected in preterm births, newborn complications, or alteration of neonatal parameters. For this reason, anti-COVID-19 vaccination should be considered safe, effective and protective of the fetus and should be promoted by all health authorities at the national and international levels.

### Strengths and Limitations

This study has confirmed, in a large population sample, previous evidence about SARS-CoV-2 vaccination in pregnancy, reinforcing its effectiveness for both mother and fetus in terms of safety and of antibody persistence/dynamics. Moreover, the antibody transfer rate in this cohort of pregnancies was well defined in terms of inclusion and exclusion criteria, clinical characteristics, and associated conditions. When analyzed according to vaccine administration, the study population appeared highly homogeneous in term of age, parity status, pregnancy pathologies, gestational age and neonatal birth at delivery.

However, some limitations need to be pointed out. Firstly, vaccination of the women was performed at different weeks of gestation. Moreover, serological data were not available for all the mother-neonate dyads, although data obtained from the vaccinated subgroup were even more precise since they were previously screened for anti-N and anti-S positive results to exclude an immune response from undiagnosed infections. Regrettably, the evaluation of specific anti-SARS-CoV-2 IFN-γ production in this study was not useful, since most of the tests resulted in indeterminate or negative results in both mothers and fetuses. However, it is known that vaccination can induce production after vaccination, as this has already been reported in the general population.

## 5. Conclusions

This study confirms and expands previous evidence on the seroconversion rate in pregnant women after SARS-CoV-2 vaccination through quantification of immunoglobulins in maternal and neonatal systemic circulation. Positive pregnancy and perinatal outcomes reinforce the evidence that immunization is safe and well-tolerated in pregnant women. Indeed, the evidence of antibody transplacental transfer reported here suggests that anti-COVID-19 vaccination is effective and protective for the fetus/neonate.

## Figures and Tables

**Table 1:** Demographic, anamnestic and clinical features of study population.

Maternal Variables	Categories	Value *n* (%)
Maternal Age	Median age (years)	34 (range: 20–48)
Ethnicity	Caucasian	191 (83.0%)
	Non-Caucasian	39 (17.0%)
Vaccination Status	Vaccinated	127 (55.2%)
	Unvaccinated	103 (44.8%)
Vaccine *	Astrazeneca	2 (1.6%)
	Astrazeneca + Pfizer	2 (1.6%)
	Moderna	6 (4.7%)
	Pfizer	112 (88.2%)
	N.A.	5 (3.9%)
Vaccine Doses *	One	9 (7.1%)
	Two	113 (89.0%)
	Three	1 (0.8%)
	N.A.	4 (3.1%)
Vaccine Side Effects *	None	23 (18.1%)
	Pain at injection site	18 (14.2%)
	Fever	3 (2.3%)
	Headache	2 (1.6%)
	N.A.	81 (63.8%)
Vaccination to Delivery Time Span *	<2 weeks	12 (9.4%)
	between 2 and 4 weeks	18 (14.2%)
	>4 weeks	87 (68.5%)
	N.A.	10 (7.9%)

Values are expressed as number and percentage. * Percentages refer to vaccinated group only (*n* = 127). Vaccination to Delivery Time Span: indicates elapsed time between last vaccine dose and maternal and neonatal plasma sampling at delivery.

**Table 2:** Vaccinated vs. unvaccinated pregnant women: maternal data and delivery, placental and neonatal outcomes.

Maternal and Neonatal Variables		UNVACCINATED *n =* 103	VACCINATED *n =* 127	*p*-Value
Maternal Age, years		33.6 *±* 5.5	34.4 *±* 4.9	ns
Maternal Ethnicity, *n*- %	*Caucasian*	72 (69.9)	119 (93.7)	*p* < 0.001
	*Not Caucasian*	31 (30.1)	8 (6.3)	
Maternal Pregestational BMI, kg/m^2^		23.5 *±* 4.8	22.16 *±* 3.4	*p* = 0.05
Gestational Age at Delivery, weeks		39.6 ± 1.1	39.6 ± 1.0	ns
Placental Weight (P), gr		563.46 ± 91.78	535.10 ± 92.16	*p* = 0.02
N/P Weight Ratio		5.9 ± 1.0	6.41 ± 1.3	*p* = 0.001
Neonatal Weight (N), gr		3279.25 ± 415.07	3344.61 ± 388.31	ns
Neonatal Weight Centiles		46.7 ± 28.8	47.6 ± 26.5	ns
Neonatal pH		7.25 ± 0.09	7.26 ± 0.09	ns
Neonatal Apgar, 1 min		9.16 ± 0.92	9.27 ± 0.82	ns
Neonatal Apgar, 5 min		9.82 ± 0.72	9.93 ± 0.31	ns

Values are expressed as mean ± standard deviation. Data were analyzed according to their distribution with independent samples using student’s *t* test, Mann-Whitney U test, or Chi-squared test for independence (with Yates continuity correction); statistical significance compared to unvaccinated group: *p* < 0.05. BMI: Body Mass Index.

**Table 3:** Serological screening to detect pre-study maternal SARS-CoV-2 infections.

	Maternal Positive Anti-N	Maternal Positive Anti-S	Cord Positive Anti-N	Cord Positive Anti-S	Total
**Unvaccinated**	13 (15.48)	5 (5.95)	3 (3.57)	3 (3.57)	24 (28.57)
**Vaccinated**	15 (13.04)	NA	1 (0.87)	NA	16 (13.91)
**Total**	28 (14.07)	5 (2.51)	4 (2.01)	3 (3.57)	40 (20.10)

Frequencies of positivity and negativity are expressed as percentages of the total number of tests.

**Table 4:** Maternal and neonatal anti-s in (**a**) all the vaccinated vs. unvaccinated subjects or in (**b**) mother-neonate dyads.

(**a**)				
		**UNVACCINATED**	**VACCINATED**	***p*-Value**
**Maternal anti-S, %**	*Positive*	0 (0%)	89 (96.7%)	*p* < 0.001
	*Negative*	49 (100%)	3 (3.3%)	
**Neonatal anti-S, %**	*Positive*	0 (0%)	84 (96.6%)	*p* < 0.001
	*Negative*	56 (100%)	3 (3.4%)	
(**b**)				
		**UNVACCINATED**	**VACCINATED**	***p*-Value**
**Maternal anti-S, %**	*Positive*	0 (0.0%)	78 (96.3%)	*p* < 0.001
	*Negative*	45 (100.0%)	3 (3.7%)	
**Neonatal anti-S, %**	*Positive*	0 (0.0%)	79 (97.5%)	
	*Negative*	45 (100.0%)	2 (2.5%)	

Frequencies of positivity and negativity are expressed as percentages of the total number of tests. Data were analyzed with Chi-squared test for independence (with Yates continuity correction); statistical significance was compared to unvaccinated group. All subjects, independently of their immunization category, were anti-N negative; among unvaccinated subjects, those who were positive for anti-S were excluded. (a) Humoral data from all the maternal and neonatal specimens, including any unpaired sample/single tests. (b) Anti-s from all mother-neonate dyads, single samples were excluded. In the vaccinated group, the two negative newborns matched with two negative mothers, while the remaining one represented the only discrepancy (negative mother and positive newborn).

**Table 5:** Maternal and neonatal plasma interferon γ (INF γ) in vaccinated vs. unvaccinated pregnant women.

		UNVACCINATED	VACCINATED
**Maternal Interferon γ**	*indeterminate*	6 (50%)	9 (39.1%)
	*non-reactive*	6 (50%)	10 (43.5%)
	*reactive*	0	4 (17.4%)
**Neonatal Interferon γ**	*indeterminate*	6 (100%)	15 (93.7%)
	*non-reactive*	0	1 (6.3%)
	*reactive*	0	0

Frequencies of reactive, non-reactive and indeterminate results are expressed as percentages of the total number of tests. Given the high number of indeterminate results, statistical significance was not reported.

## Data Availability

All data supporting the findings of this study are available from the authors on reasonable request.

## Supplementary Materials

The following supporting information can be downloaded at: https://www.mdpi.com/article/10.3390/jpm13040689/s1, Table S1: Details of all subjects analyzed for T-cell mediated immune response.
