# Supplementary material for: Immune Response and Transplacental Antibody Transfer in Pregnant Women after COVID-19 Vaccination
Source: J Pers Med. 2023 Apr 20;13(4):689. doi: 10.3390/jpm13040689 (PMC10141882; doi:10.3390/jpm13040689)
Supplement: Supplementary file 1 [file jpm-13-00689-s001.zip › jpm-2264942-supplementary.pdf]

Table S1. Details of all the subjects analyzed for T-cell mediated immune response

Study ID	Dose-Sampling Interval	Anti-N Interpretation	Anti-S Interpretation	Anti-S Interpretation	Maternal IFN-γ	Fetal IFN-γ
BU120C39b	NA	NEG	NEG	NEG	NonReactive	Indeterminate
BU122C40a	NA	NEG	POS	POS	Indeterminate	Indeterminate
BU123C40b	NA	POS	NA	NA	Indeterminate	Indeterminate
BU125C41a	NA	NEG	NEG	NEG	Indeterminate	Indeterminate
BU126C41b	NA	NEG	NEG	NA	Indeterminate	Indeterminate
BU128C42a	NA	NEG	NEG	NEG	Indeterminate	Indeterminate
SA002C1a	NA	NEG	NEG	NA	NonReactive	NA
SA002C1b	NA	NEG	NEG	NEG	NonReactive	NA
SA004C2a	NA	NEG	POS	NA	NonReactive	NA
SA004C2b	NA	NEG	NEG	NEG	NonReactive	NA
SA006C3a	NA	POS	NA	NA	NonReactive	NA
SA006C3b	NA	NEG	NEG	NA	Indeterminate	NA
BU154V51	21	NEG	NEG	POS	Indeterminate	Indeterminate
BU157V52	112	NEG	POS	NA	Indeterminate	Indeterminate
BU160V53	79	NEG	POS	POS	Indeterminate	NonReactive
BU169V56	64	NEG	POS	POS	NonReactive	Indeterminate
BU172V57	113	NEG	POS	POS	Indeterminate	Indeterminate
BU175V58	285	POS	POS	NA	Indeterminate	Indeterminate
BU184V61	NA	NEG	POS	NA	Reactive	Indeterminate
BU190V63	146	NEG	POS	POS	Indeterminate	Indeterminate
BU193V64	107	NEG	POS	POS	Indeterminate	Indeterminate
BU196V65	118	POS	NA	NA	NonReactive	Indeterminate
BU199V66	88	NEG	POS	POS	NonReactive	Indeterminate
BU202V67	28	NEG	POS	POS	Reactive	Indeterminate
BU205V68	98	POS	NA	NA	Indeterminate	Indeterminate
BU208V69	140	NEG	POS	POS	NonReactive	Indeterminate
BU211V70	102	NEG	POS	POS	NonReactive	Indeterminate
BU213V71	133	NEG	POS	POS	NonReactive	Indeterminate
SA001V1	NA	NEG	POS	NA	Indeterminate	NA
SA003V2	1	POS	NA	NA	NonReactive	NA
SA005V3	119	NEG	POS	NA	NonReactive	NA
SA007V4	NA	NEG	POS	POS	Reactive	NA
SA009V5	NA	NEG	POS	POS	NonReactive	NA
SA011V6	58	NEG	POS	NA	NonReactive	NA
SA013V7	12	NEG	POS	POS	Reactive	NA
